# A New Model Supporting Stability Quality of Materials and Industrial Products

**DOI:** 10.3390/ma15134440

**Published:** 2022-06-23

**Authors:** Dominika Siwiec, Andrzej Pacana

**Affiliations:** Faculty of Mechanical Engineering and Aeronautics, Rzeszow University of Technology, al. Powstancow Warszawy 12, 35-959 Rzeszow, Poland; d.siwiec@prz.edu.pl

**Keywords:** grey relational analysis, Ishikawa diagram, multi-criteria decision methods, quality management tools, production engineering, mechanical engineering

## Abstract

Stabilizing the quality of industrial product materials remains a challenge. This applies mainly to new or significantly modified materials. It also refers to special processes. The tests of product quality can stabilize the quality of industrial product materials. The popular method for this is using the non-destructive testing (NDT). The NDT identifies incompatibility but does not determine the cause of its occurrence. Hence, it was necessary to support the process of identifying causes of incompatibilities in products. The purpose of the article was to develop a model based on a new approach to determine the ranking of actions that are possible as part of the process of stabilizing the quality of industrial products. The model was developed to improve quality through sequential and systematic methods of identification (and reduce) and incompatibility. The quality management techniques and decision method were applied and combined in this model, i.e., SMART(-ER) the method, method of selecting a team of experts, brainstorming (BM), Ishikawa diagram with the 5M rule, Likert scale validation technique, arithmetic average, and Grey Relational Analysis (GRA). The test of this model was carried out to find cracks in the outer hull of 418 alloy four-point bearing (CPW-S 5616), which was identified by NDT (magnetic-powder method). As a result, a ranking of activities was obtained to stabilize the quality of the product and the main cause of incompatibility was indicated, i.e., the cause which can influence to the most degree influence on occurrence the incompatibility. The originality of the proposed model is an application in the right order of specially selected and combined qualitative methods and supporting decision methods. The finding of causes of incompatibility of products is the basis of product improvement in the area of stabilizing the quality of materials, mainly by the occurrence of special processes. The universality of the model refers to the possibility of its application for any material, processes of its formation, and processes of products, and any incompatibilities where the model can be integrated with quality control.

## 1. Introduction

The special processes of materials are difficult to stabilize. Special processes are realized based on the validated process (method or algorithm). The identification and removal of these incompatibilities improve manufacturing quality or a quality of the processes as the method. The research of the quality of materials and products is mainly non-destructive testing [1]. However, this research does not determine the causes of incompatibility. Therefore, it is necessary to take future actions which rely on determining the causes of these incompatibilities [2,3,4]. In the process of achieving high quality of products [5], it is necessary to identify and eliminate possible incompatibilities of products [6,7]. It refers to determining the main causes of the problem in a precise way to carry out adequate improvement actions [8,9]. The right analysis of the quality of products and the implementation of supporting actions to reduce incompatibilities also contribute to stabilizing the production process [10,11]. Furthermore, it is possible to repeat results under industrial conditions [5,12,13]. Despite that, achieving stable production is still problematic [14,15]. The initial production series is carried out mainly in the case of new products or significantly modified [16].

The literature review shows that to verify incompatibilities of industrial products, the most frequently used methods were brainstorming (BM), Ishikawa diagram [17], Pareto-Lorenz [18], and the 5Why method [19]. The Ishikawa diagram and the Pareto-Lorenz diagram were used to verify the causes of incompatibility of industrial products [18,19,20,21]. For example, in study [18], the structure of the pulley defects was assessed, where the Pareto diagram was used to identify the most important incompatibilities, and the Ishikawa diagram was used to determine the causes of these incompatibilities. In turn, the study [22] presented a combination of methods, i.e., the Ishikawa diagram and Pareto analysis, to reduce defects of capacitors. Firstly, all defects were verified by causes and effects diagrams, and then the most important defects were identified by Pareto analysis. In a similar way, the authors of the study [19] analyzed the laser cutting process, where the 5Why method was used to verify the main causes of incompatibility in this process. However, in study [20], the general purpose technological analysis (GPT) was verified using the Ishikawa diagram. The universalmodel was carried out by authors of study [12], in which quality management tools were combined, that is, the SMART (-ER) method, the method of selecting a team of experts, brainstorming (BM), Ishikawa diagram, and the 5Why method. The model was carried out by mechanical seal from 410 alloy. Another example is in this study [21], in which the Ishikawa diagram was applied to analyze the causes of errors in assessment accuracy in part of the construction of the machine. The authors of this study presented another approach to the analysis of the incompatibility of the product [23], where the incompatibility of metal inclusions in the product from AMS6514 alloy was verified. The techniques were combined, i.e., brainstorming, Ishikawa diagram, and DEMATEL method. The idea was to verify cause-and-effects relations of incompatibility of product. In the study [24], the method was tested that was a combination technique, that is, brainstorming the Ishikawa diagram and the DEMATEL method. The purpose was to develop combination methods to verify complex cause-and-effect problems. These problems refer to the quality of products. The number of causes of these incompatibilities was large. These methods were shown in the study [25,26,27], where the techniques were combined: brainstorming (BM), cause and effect diagram, FAHP method (Fuzzy Analytic Hierarchy Process), and 5Why method (Why-Why method). The idea of this combination was to reduce inconsistencies and uncertainties in the expert team, where these evaluations refer to a large number of causes of product incompatibility. In turn, in this study [28], practical examples of using the basic quality tools (7QC tools) were analyzed. Other causes were shown in the study [29], in which there were analysis groups (categories) of the causes of problem according to the Ishikawa rule (5M+E). This tool was combined with the FTOPSIS method (Fuzzy Technique for Order Preference by Similarity to Ideal Solution) and the FAHP method. The test method was carried out to determine cracks in the pin connecting discs in the engine gear when grinding. The purpose of this combination was to verify the causes of various types of incompatibility of products.

It was concluded that quality management tools were used to verify the incompatibilities of industrial products, e.g., [12,18,21]. Their applications include verifying the potential causes of incompatibilities and determining the main causes [22,23]. Despite that, these analyses were not a destination of sequential reducing causes of incompatibility by its importance, i.e., determining the impact (importance) of these causes on occurring incompatibility. Therefore, these studies include a gap in the lack of methods that are applied to create a ranking of causes of incompatibility, where this ranking would allow for ranking actions that support the stabilization of product quality. Therefore, the objective of the article was to develop a new model that supports the stabilization quality of industrial products. During the development of this model, the hypothesis was assumed:

**Hypothesis** **1** **(H1).** *It is possible to support the stability quality of materials, processes of their formation and industrial products by determining the ranking of causes that have an impact on the incompatibility of the product; it is realized by determining all potential causes and then by their sequential and constructive reduction to reduce the main causes.*

The purpose of the study is to develop a model based on a new approach to determine the ranking of actions that can be taken as part of the process of stabilizing the quality of industrial products. It refers to the sequential and coherent analysis of the causes of problems with the quality of products, where the analysis can be realized even for four causes of incompatibility. It means that it is possible to analyze a small number of causes of incompatibility, where according to the GRA method, the minimum number of causes is equal to 4. However, the maximum number of causes of incompatibility for analysis is unlimited. Moreover, it is possible to reduce the causes of the problem from potential causes to the most important causes, which possibly have the largest degree of influence on the occurrence of the problem.

The model has a universal character. However, in view of the specificity of special processes occurring in the mechanical industry, it seems adequate to realize the initial test, for example, for the casting process. That resulted from a review of the literature on the subject [18,19,20,21,24,26,29], after which it was proved that the most frequent errors occur due to foundry processes. Therefore, the modeling was tested for cracks in the outer hull of the four-point bearing of alloy 418 (CPW-S 5616). This incompatibility was identified in the Polish company by non-destructive testing (magnetic powder method).

## 2. Model

### 2.1. Concept of Model

**Figure 1:**
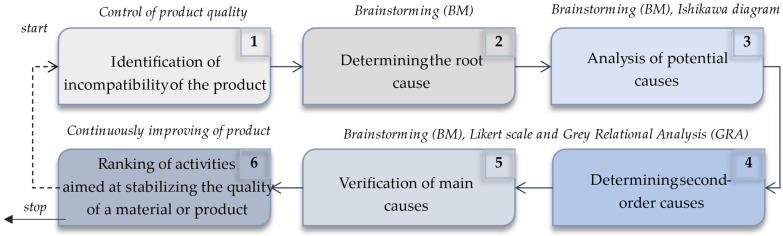
General concept of model.

The concept of the model refers to the verification of incompatibility of industrial products, where efforts were made on systematic verification of incompatibilities as part of continuous improvement of products. The idea was to support the production process of products, i.e., mainly new products or modified products for which often the trial (initial) production series are realized. The main purpose of the model was to determine the ranking of actions that stabilize the quality of products. The general concept of the model is shown in Figure 1.

The model was developed as a modified method and suited to the specification of searching for the importance of incompatibilities in industrial products. The mentioned support was a destination in the process of identification of incompatibility causes and their importance in determining the main causes of the problem, after which it is possible to make the right improvement actions. It refers to verification of all potential causes of incompatibility, their sequential analysis, and reducing to identify the main causes (i.e., having the most impact on the emergence of noncompliance). After determining the main causes of incompatibility, adequate improving actions are determined, i.e., actions which can reduce or mostly reduce incompatibility.

The originality of the model is the possibility to analyze, in sequential and coherent ways, the causes of incompatibilities of the product, where the number of these incompatibilities can be equal even to 4. Moreover, it is possible to reduce causes from potential causes to the most important causes, where this process is supported by a calculation process and a simple but simultaneously effective GRA method. Additionally, the model combines techniques: teamwork, visualization, and calculation methods, which support and realize each step of the model to identify the main cause (root) of problems.

### 2.2. Conditions and Justification for Choice of Methods for Model

The concept of the model is based on the integration of selected instruments of quality management and decision support methods in a fuzzy decision-making environment. These techniques were: SMART(-ER) method [30], method of choice of the team of experts [12,31], brainstorming (BM) [32,33], Ishikawa diagram (causes and effects) with the 5M rule [6,7,8,9], the technique of importance in Liker scale [5,7], average arithmetic, and Grey Relational Analysis (GRA) [34,35,36,37,38,39]. 

Firstly, the purpose of the analysis is determined. The purpose is determined by the SMART(-ER) method (S—specific, M—measurable, A—achievable, R—relevant or realistic or reward, T—based on timeline or timebound, E—exciting or evaluated, R—recorded or reward) [30].

Next, the team of experts is selected. The team is selected according to the method shown in the study [12,31]. The idea was to achieve an effective analysis of a problem by the team of experts. Therefore, the expert team should have knowledge and experience in the analysis of incompatibility and the ability to solve the problem. The appropriate choice of the team of experts has an impact on achieving the objective, as shown in the study [9].

Next, brainstorming (BM) is realized among a team of experts. The BM method allows for coherent and effective verification of any kind of problem that requires in-depth analysis [32,33]. This method is used in all stages of the proposed model, for example, to identify the potential causes of incompatibility (i.e., the causes which probably cause incompatibility of product). To determine these causes, it is necessary to answer the question “What has happened that this incompatibility occurred?”.

Next, all causes are grouped according to the 5M rule (man, method, machine, material, and management). This rule is preferred to analyze the quality of industrial products [19,20]. The 5M is used in the Ishikawa diagram to group the causes of the problem. The aim of 5M is the simple visualization of the causes of incompatibility of products [21]. Therefore, the team of experts needs to understand each of the causes generated during brainstorming (BM). 

Then, the team of experts determines the weights of incompatibility causes, i.e., the impact of these causes on the occurrence of incompatibility. This is achieved as part of the next part of brainstorming (BM) and by using the technique with the Likert scale to determine the importance of causes [5,7]. According to these assessments, the second-order causes of incompatibility are determined, that is, the ones that have the most impact on the occurring incompatibility from all potential causes. It refers to the estimate of the weights of causes according to arithmetic average from assessments of potential causes. The presentation of the weights of causes as average values of the assessments of the team of experts resulted from the need to combine all the evaluations as a single value. It is difficult and not precise to compare the causes when they are marked by a large number of different assessments.

Later, in a combined way techniques are used such as brainstorming (BM), importance technique with Likert scale, and GRA method. The purpose is to determine the main causes, i.e., having the maximum weight, the most impact on incompatibility. The GRA method has application for a small number of data (that is, even 4 data), where it is a common phenomenon during the analysis of causes of incompatibility [38,39].

### 2.3. Model Assumptions and Conditions Ensuring Its Novelty

The assumptions of the model were made after making the concept of the model and determining the conditions of the selected techniques. Moreover, these assumptions have resulted from literature review, e.g.:

- a lack of limitations for the number of potential causes [2,19];
- the minimum number of potential causes determined for a single category (5M) in the Ishikawa diagram is equal to 4 causes [36,37,39];
- the minimum number of all second-order causes in the Ishikawa diagram should be equal to 4 causes [34,35,39];
- Second-order causes are causes having the greatest impact on incompatibility of all potential causes [12,38];
- the main causes are the causes that have the most impact on incompatibility from all second-order causes [20,23];
- the causes of potential incompatibility may or may not be of equal importance to each other, i.e., they may have the same or different influence (severity) on the occurrence of incompatibility [12,39];
- verification of the impact of causes on occurred the incompatibility is supported by the process of importance of causes on the Likert scale by selected teams of experts [12,38].

These assumptions were detailed in stages of model, which was characterized in the next part of the study. The novelty of the model is possibilities of its application for any product, e.g., the new production process (new products) or a significantly modified product, where this production is not stable, for example, starting a trial (initial) series. Additionally, the model has applications for any kind of incompatibilities identified as part of special processes. Therefore, the model can be used for any entity (e.g., production enterprise). Furthermore, the model can be integrated with any quality control after which incompatibility was identified [12]. The novelty of the model resulted from the character of the implemented quality management tools and decision methods, e.g., the possibilities to analyze even four incompatibilities [34,35]. Therefore, the model can be used as part of continually improving products, and for the sustainable development of industrial products.

### 2.4. Characteristics of Model

**Figure 2:**
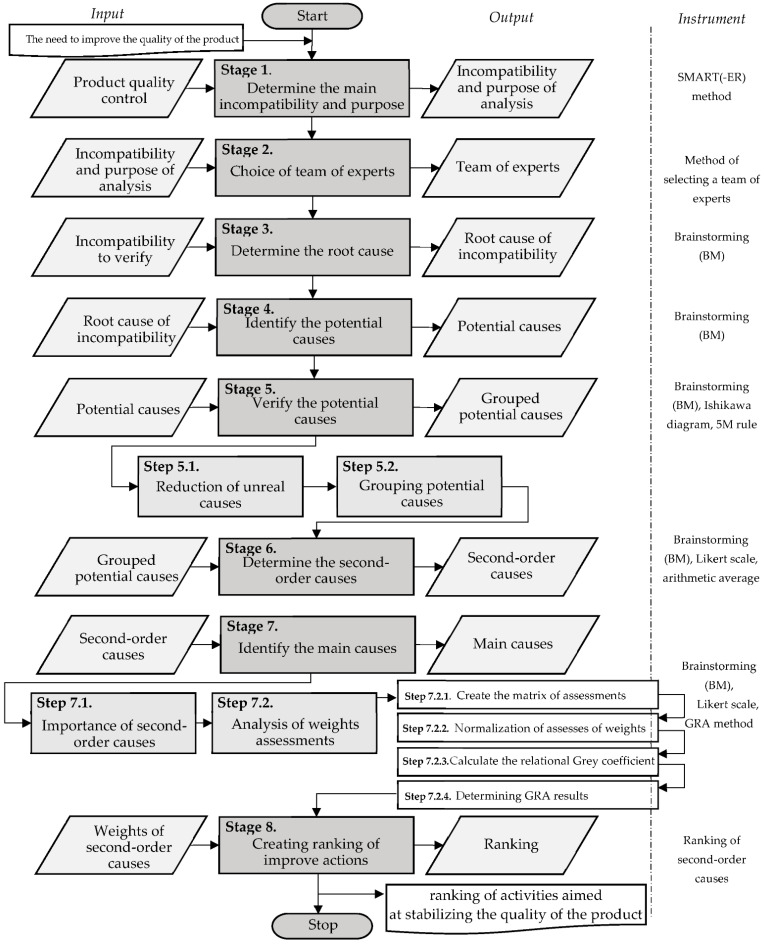
Model supporting stabilization quality of industrial products.

The purpose of the proposed model is to support the stabilization of the quality of industrial products. The model was developed in eight main stages, as shown in Figure 2.

Detailed characteristics of the model stages are presented in the next part of the study.

**Stage 1**. Determine the main incompatibility and purpose of the analysis

The incompatibility to analyze should be the main incompatibility, i.e., incompatibility which is the most often occurring in the enterprise and has the biggest cost or affected waste of resources. This incompatibility is determined according to the control sheet or using Pareto analysis [18]. Then, for the chosen incompatibility, the purpose of the analysis is determined. The SMART(-ER) method is used for that [30]. The purpose is determined by the entity, e.g., an expert (for example, a quality control manager or a company owner). The incompatibility should be characterized by considering, e.g., the type of incompatibility, the product in which this incompatibility was identified, and the number of incompatibilities. This information about incompatibility is often available in the catalogue (specification) of incompatibility.

**Stage 2.** Choice of the team of experts

The purpose of choosing a team of experts is to determine the people responsible for executing the model and achieving the purpose of the model. This incompatibility of the product (selected at stage 1) should be analyzed by the expert team to precisely determine the main cause of this incompatibility. Therefore, the expert team should have knowledge and experience in the analysis of incompatibility and the ability to solve the problem. The appropriate choice of the team of experts has an impact on achieving the objective, as shown in the study [9]. The team of experts should be chosen according to the method shown in the study, i.e., [12,31].

**Stage 3.** Determine the root cause

Determining the root cause refers to determining the incompatibility at the place of occurrence. To determine the root cause brainstorming (BM), it is used, which is shown among the team of experts. The Pareto rule (20/80) can be used for a large number of root causes, as shown in the study [18,20].

**Stage 4.** Identify the potential causes

Identification of potential causes (initial causes) includes determining causes that could have an impact on occurring incompatibility. At this stage, all potential causes are determined. Their impact (weight) on the incompatibility will be determined in the next stage of the model. To determine these causes, it is necessary to answer the question: “What has happened that this incompatibility occurred?”. To identify potential causes, brainstorming is conducted among the team of experts. The brainstorming is carried out according to the method, which is shown in the study, e.g., [32,33]. It is necessary to generate (indication) all potential causes based on the root of the incompatibility (from stage 3). All potential causes are noted in a place visible to the team, e.g., a blackboard. The BM should end after about 30 min. The result of this stage is the highest number of potential causes of incompatibility.

**Stage 5.** Verify the potential causes

At this stage, verification of all potential causes is done. The purpose of this stage is to determine the potential causes, which could have an impact on the occurred incompatibility, and then show these causes in a standardized way. The result of this stage is the Ishikawa diagram for potential (initial) causes, which are grouped according to the 5M rule [18,19,21]. At this stage, all potential causes have the same weights, i.e., impact into incompatibility.

*Step 5.1. Reduction of unreal causes*

Firstly, it is necessary to delete potential causes which are unreal, i.e., that probably do not have an impact on incompatibility. For this purpose, the leader analyzes all potential causes (generated on stage 4) and, from among them, removes unreal causes.

*Step 5.2. Grouping potential causes*

Potential causes are grouped according to their categories. It was assumed to use the 5M rule, i.e., man, method, machine, materials, and management. However, it is possible to use any category which will be adequate for determining potential causes, for example, personnel measurement, environment. Brainstorming is used to group these causes, which is done among the selected team of experts. In turn, the Ishikawa diagram is used to group potential causes [18,19]. Next, for each category (5M), it is necessary to note appropriate potential causes. According to the concept of the model, in the Ishikawa diagram, it is necessary to note a minimum of four potential causes in each category of 5M [35,37].

**Stage 6.** Determine the second-order causes

At this stage, the weights (importance) of the potential causes were determined. It refers to determining the impact (importance) of these causes on incompatibility. Therefore, it is determined which potential causes could have the most likely to cause the incompatibility. Determining second-order causes relies on analyzing potential causes in each group of these causes (i.e., 5M) and determining their importance (impact) on the occurrence of the incompatibilities. For this purpose, it is necessary to use, in a combined way, brainstorming (BM), Likert scale, and arithmetic average from weight assessment. As a result, the ranking of potential causes is obtained, where the maximum weight in each group of 5M is the second-order cause. The team of experts assesses potential causes, i.e., determines importance (weight) of the impact of potential causes of incompatibility. In this aim, the brainstorming is carried out during which a team of experts assesses causes on the Likert scale [5,7], where 1—the cause has little influence on incompatibility (low importance), 5—the cause significantly influences the occurrence of incompatibility (high importance). All potential causes shown on the Ishikawa diagram (from the 5 stage) should be assessed. The assessments are noted directly on the Ishikawa diagram by potential causes. The assessments are noted directly on the Ishikawa diagram by potential causes. After assessing all potential causes, it is necessary to estimate the weights of these causes. The arithmetic averages of all assessments of the weights are calculated (1):

(1) $$w_{i}=\frac{\sum_{i=1}^{n}w_{i}}{n}$$

where: *w*—weight *i*-th potential cause, *n*—number of evaluates for *i*-th potential cause.

Second-order causes are chosen on the average weights of potential causes. It is necessary to choose a single cause in each group of causes (i.e., from each of 5M groups). It is a cause that has a maximum average weight value. It is useful to note these causes in the Ishikawa diagram. The second-order causes are verified in the next stage of the model.

**Stage 7.** Identify the main causes

At this stage, second-order causes are verified. The purpose is to identify the main causes, i.e., causes that have the most impact on the occurrence of incompatibility. It refers to reverifying second-order causes. Hence, these causes are verified simultaneously (without including their groups). In this purpose, it was assumed to be used in a combined way: brainstorming (BM) [40,41], Likert scale [5,7], and GRA method [35,38]. As a result, the second-order causes are achieved, where the maximum weight is the main cause. If some second-order causes will have the same weight, these causes are considered equally important. It is shown in three steps.

*Step 7.1. Importance of second-order causes*

The team of experts assess second-order causes, i.e., determines importance (weight) impact of causes on the occurrence of incompatibility. In this aim, the brainstorming is carried out during which a team of experts assess causes on the Likert scale [5,7], where 1—the cause has little influence on incompatibility (low importance), 5—the cause significantly influences the occurrence of incompatibility (high importance).

*Step 7.2. Analysis of weight assessments of second-order causes*

Based on weight assessments of second-order causes, it is necessary to verify their importance. The GRA method is used for this [37,38,42,43]. The choice of the GRA method resulted from its application to supporting decisions in the fuzzy (uncertain) area [34,35], where it is adequate to verify the importance of causes of incompatibility causes (determined subjectively by team of experts [36,37]. Furthermore, the GRA method has application for a small number of data (i.e., even 4 data), where it is a common phenomenon during analysis of causes of incompatibility [38,39], except that it conditioned versatility of the proposed model, where it is possible to verify both a large and a small number of causes. Ultimately, it is possible to determine adequate improvement actions. The method is shown in four steps. The result of this stage is weights of second-order causes.

Step 7.2.1. Create the matrix of assessments

First, the matrix is created, i.e., M=m×n, where *m*—alternative (that is, group of causes), *n*—criterion (that is, cause). This matrix should be filled with weights of second-order causes [34,35].

Step 7.2.2. Normalization of assessment of weight of incompatibility causes

Then, it is necessary to process (normalize) the weights of causes to achieve assessments in the range from 0 to 1. According to the concept of a model, it was assumed that “the higher the rating, the greater the impact of the cause on the incompatibility”. Therefore, according to the GRA method, Formula (2) is used [34,37]:

(2) $$x_{i}^{*}\left(k\right)=\frac{x_{i}^{\left(O\right)}\left(k\right)-min\;x_{i}^{\left(O\right)}\left(k\right)}{max\;x_{i}^{\left(O\right)}\left(k\right)-min\;x_{i}^{\left(O\right)}\left(k\right)}$$

It was assumed that $x_{0}^{\left(O\right)}\left(k\right)$ and $x_{i}^{\left(O\right)}\left(k\right)$ are appropriately original and comparable sequence; i=1, 2, …, m; k=1, 2, …, n; and *m*—alternative (i.e., group of causes), *n*—criterion (i.e., cause) [3,43].

Step 7.2.3. Calculate the relational Grey coefficient

On the basis of normalized sequences, the relational Grey coefficient is calculated. Formula (3) is used for that [36,37]:

(3) $$\gamma \left[x_{0}^{*}\left(k\right),\;x_{i}^{*}\left(k\right)\right]=\frac{\Delta_{min}+\xi \Delta_{max}}{\Delta_{0i}\left(k\right)+\xi \Delta_{max}},\mathrm{and}\;0<\gamma \left[x_{0}^{*}\left(k\right),\;x_{i}^{*}\left(k\right)\right]\leq 1$$

where: $\Delta_{0i}\left(k\right)$ represents a sequence of variations between the original sequence $x_{0}^{*}\left(k\right)$ and comparison sequence $x_{i}^{*}\left(k\right)$, which is calculated from Formula (4) [39]:

(4) $$\Delta_{0i}\left(k\right)=|x_{0}^{*}\left(k\right)-x_{i}^{*}\left(k\right)|$$

Similarly, the largest (5) and the smallest (6) deviations are calculated [34,35,36]:

(5) $$\Delta_{max}=\underset{\forall j\in i}{\max}\;\underset{\forall k}{\max}\left|x_{0}^{*}\left(k\right)-x_{j}^{*}\left(k\right)\right|$$

(6) $$\Delta_{min}=\underset{\forall j\in i}{\min}\;\underset{\forall k}{\min}\left|x_{0}^{*}\left(k\right)-x_{j}^{*}\left(k\right)\right|$$

In turn, the factor ξ from Formula (3) has values [0,1]. Most often, it is assumed that ξ = 0.5 [35,38].

Step 7.2.4. Determining Grey Relational Assessments

The relationship assessment of Grey is the weighted sum of Grey’s coefficients, as shown in Formula (7) [36]:

(7) $$\gamma \left(x_{0}^{*},\;x_{i}^{*}\right)=\overset{n}{\underset{k=i}{\sum }}\beta_{k}\gamma \left[x_{0}^{*}\left(k\right),\;x_{i}^{*}\left(k\right)\right]$$

where: $\gamma \left(x_{0}^{*},\;x_{i}^{*}\right)$, *tj*. a grey relational score that shows the level of correlation between the original sequence and the comparable sequence, as if they were identical.

A correctly defined grey relational score should be 1 (8) [34,40]:

(8) $$\overset{n}{\underset{k=1}{\sum }}\beta k=1$$

As a result, on the basis of the GRA results, it is possible to determine the degree of influence of the second-order causes on the occurrence of incompatibility. This is shown in the next step of the model.

*Step 7.3. Choice of main cause*

Based on calculated values, it is possible to choose the main cause which has the largest impact on incompatibility. It is the cause that has the maximum value according to the GRA method [36]. It is useful to mark this cause in the Ishikawa diagram.

**Stage 8.** Creating a ranking of actions as part of stabilizing the quality of the product

This stage relies on ordering the weight values of second-order causes (estimated in Step 7.2). The cause with the maximum GRA value is the main cause (the first position in the ranking). The lower the value of GRA, the lower the cause is. According to the rules for continuous improvement of products [2,3,4], in the first position, it is necessary to propose improvement actions for the main cause. Following the authors of [18], eliminating the root cause in the first place can ensure that the incompatibility is reduced or eliminated with a certain probability. After improvement actions, it is necessary to verify their efficiency [40,41]. Then, it is possible to take actions for other incompatibilities of ranking. For that, it is necessary to use the sequence developed in the proposed model. Actions implemented in this way (in a repeatable and sequential manner) will help stabilize the quality of the product as part of the continuous improvement process.

## 3. Test of Model

The model was tested for cracks on the outer hull of the 418 alloy four-point bearing (CPW-S 5616), which were identified relatively often in the Polish company. The Polish industry was chosen because the foundry industry is the most developed in Poland. Additionally, in the foundry industry, special processes are the most needed, as shown in the studies [18,19,20,21,24,26,29]. Cracks occur as a result of the loss of ductility of the material (i.e., exceed the tensile strength). In enterprises, the methods supporting accurate determination are the main causes of incompatibility. So far, the bearing verification of the incompatibility has been based mainly on the experience and decisions of the quality manager. The choice of the outer hull of the 418 alloy four-point bearing was based on the individual needs of the enterprise. The incompatibilities of this product were verified relatively often, and the main causes of its occurrence were not precisely identified. In addition, in the general approach, the four-point bearing is popular and often used, for example, in the engineering industry. Therefore, the results obtained for this product can be useful in different applications. For this reason, it was considered justified to propose a model that supports the stabilization of the quality of this product.

**Stage 1.** Determine the main incompatibility and purpose of the analysis

**Figure 3:**
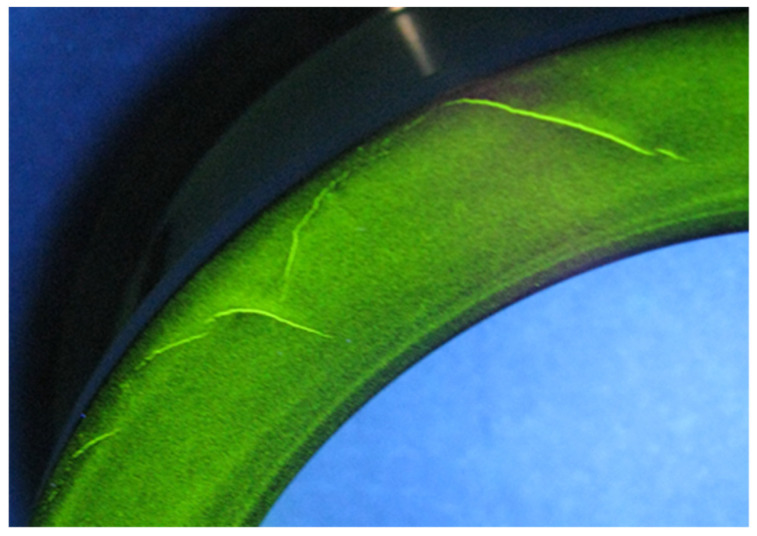
Crack on the outer hull of four-point bearing.

The main incompatibility was a crack in the outer hull of 418 alloy four-point bearing (CPW-S 5616). This incompatibility was identified by non-destructive tests (magnetic powder method), as shown in this study [2]. The crack was a flat incompatibility, where stresses occur at the ends of it. As a result, a notch will form, causing the crack to develop further [42]. The example of the crack in the outer hull is shown in Figure 3.

The outer hull of the four-point bearing was specially designed for oil-free screw compressors. They have a high-strength outer cage ring and can be operated accurately. These products can reach high speeds under high operating temperatures and high loads. In addition, they provide reduced heat, vibration, and noise generation. The outer four-point bearing hull is used, among others, mounted on the trunnion of the first rotor disc of the compressor [43,44].

The outer hull of bearing was made as forging from 418 (CPW-S 5616) alloy. It is an alloy of modification with higher strength from the family of 12% chrome martensitic stainless steels. It is a precipitation hardening stainless steel with shear strength. Its chemical composition is as follows: Fe (81%), Cr (13%), W (3%), Ni (2%), and C (1%). The mechanical and physical strengths are presented in Table 1.

Based on the selected incompatibility, it was possible to determine the purpose of the analysis. The purpose was to determine the rank of actions to stabilize quality of the outer hull of bearing from 418 alloy. This concerned the sequential reduction and the importance of the causes of fracture in the bearing housing until the main causes of this problem were identified and the sequence of appropriate improvement actions could be determined.

**Stage 2.** Choice of the team of experts

According to the expert second stage, the team was selected. The team included a quality control manager, NDT manager, and authors of the article. The team of experts had knowledge and experience in this type of incompatibility and methods used in the proposed model.

**Stage 3.** Determine the root cause

At this stage, the root cause of the crack on the outer hull of the four-point bearing was determined. For this purpose, brainstorming was conducted among the experts’ team. It was assumed that root cause of the crack is a state of stress (deformation). As a result, the tensile strength of the material is locally exceeded. This is called loss of ductility and is followed by the formation of a notch that generates this incompatibility. This defect may cause the product to crack during operation.

**Stage 4.** Identify the potential causes

Then, the potential causes (initial) of the crack were identified. For this purpose, brainstorming was conducted among the experts’ team. Potential causes such as:

1. Too fast welding speed;
2. Flow of liquid weld pool too fast;
3. Lack of clean welded layer;
4. Small width in relation to depth (joint proportion);
5. Stresses due to high thermal expansion;
6. Inappropriate selection of material;
7. Unprepared metal surface;
8. High carbon content in the weld;
9. Dirt inside the weld;
10. Employee rush;
11. No periodic training;
12. No TMP (Total Productive Maintenance);
13. Lack of up-to-date procedures;
14. Distraction;
15. Dirty tools;
16. Lack of unit controls;
17. Broken tools;
18. Failure to use the manual;
19. Moisture of the electrode;
20. Short work experience of the employee;
21. Inadequate lighting;
22. Noise;
23. Contamination of the site;
24. Uncalibrated tools;
25. Psychophysical condition of the worker (e.g., severe nervous tension, exhaustion, physical or mental malaise).

Twenty-five potential causes were identified and subjected to further verification in subsequent stages of the model.

**Stage 5.** Verify the potential causes

At this stage, all potential (initial) causes were verified. The purpose was to determine potential causes that could have an impact on incompatibility.

*Step 5.1. Reduction of unreal causes*

First, the impossible cause was deleted from all 25 potential causes, i.e., dirty tools. The result was the general overview of the tools used, which were kept in an orderly manner. Other causes were determined as possible causes of the outer hull of the bearing, which was made as forgings from alloy 418 (CPW-S 5616).

*Step 5.2. Grouping potential causes*

**Figure 4:**
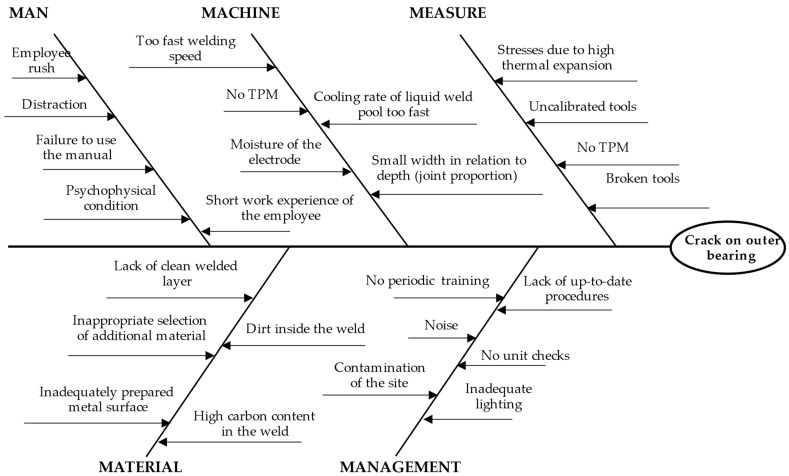
Ishikawa diagram for a crack in the bearing housing.

Then, the potential causes were grouped according to Rule 5M, i.e., man, method, machine, material, and management. Causes were grouped during brainstorming (BM) among a team of experts. The Ishikawa diagram was created, as shown in Figure 4.

The developed Ishikawa diagram was modified after the next stages of the model were implemented.

**Stage 6.** Determine the second-order causes

At this stage, the team of experts determined the impact (importance) of potential causes. For this purpose, all causes from the Ishikawa diagram were analyzed. The impact (weight) of potential causes was determined in each 5M group. The brainstorming scale, Likert scale, and arithmetic average were used in a combined way.

**Figure 5:**
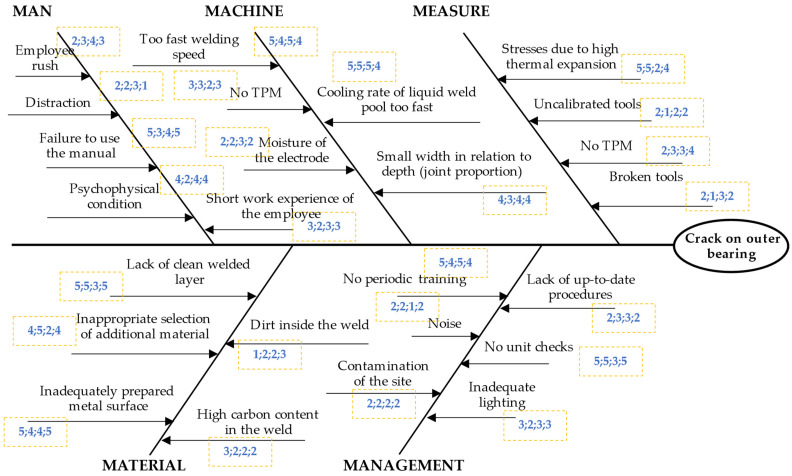
Ishikawa diagram for crack on the outer hull of four-point bearing to assess the importance of potential causes.

The team of experts assessed the potential causes, i.e., the importance of potential causes of cracks on the outer hull of bearing. During brainstorming (BM), causes were assessed on the Likert scale. These assessments are shown in Figure 5.

Then, it was possible to verify the causes and their assessments. The analysis included assessments of the weights of causes in the 5M group. The average of the assessments was calculated as the average weight of potential causes. The result is shown in Table 2.

It is not certain that we have identified all causes, therefore, the sum of average values should not exceed 100%. In this case, the value was equal to about 80% and is acceptable.

**Figure 6:**
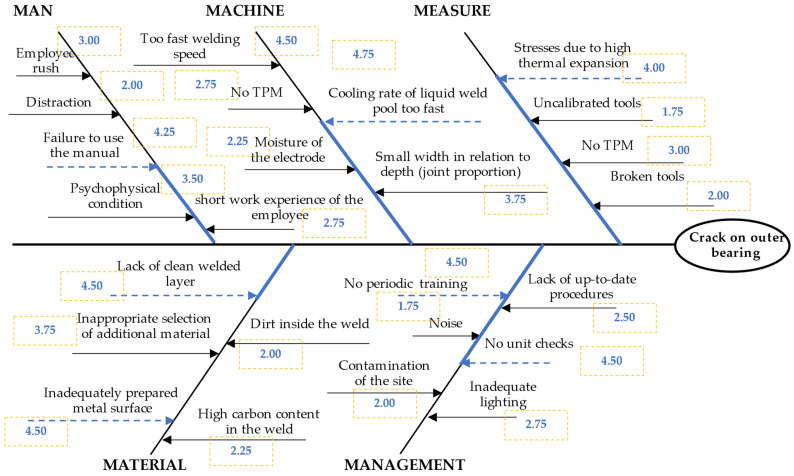
Ishikawa diagram for crack on the outer hull of four-point bearing to choose the second-order causes.

Second-order causes were selected based on the basis of average weights of potential causes. Second-order causes were selected in each group of causes (i.e., from each group of 5M). These were causes with the highest value of the arithmetic average. Second-order causes were not following the manual (C3), too high cooling rate of the liquid weld pool (C9), stresses (C11), no cleaned surface layer (C15), inadequately prepared metal surface (17), no periodic training (20), and no unit check (C24). The average values resulting from the assessments awarded by a team of experts (during the brainstorming method before step) were marked in the Ishikawa diagram (Figure 6).

The main causes from second-order causes were selected as the main causes. It is shown in the next stage of the model.

**Stage 7.** Identify the main causes

Initially, the team of experts assessed second-order causes, i.e., determined the importance (weights) of the impact the causes on cracks on the outer hull of four-point bearings. Brainstorming was used for that. The importance of second-order causes was assessed using the Likert scale. The result is shown in Table 3.

For the assessment of weights of second-order causes, it was achieved according to the GRA method. This included phases 1 to 4 of the model. First, the average assessments of Table 4 were normalized. Formula (2) was used for this. Then, with normalized sequences, the relational Grey coefficient was calculated. Formula (3) is used for those and adequate Formulas (4)–(6). The Grey Relational Assessment (GRA) is the weighted sum of the Grey coefficients, as shown in Formula (7). Based on GRA values, the ranking was created. The maximum value is determined as the main cause. The results are shown in Table 4.

**Figure 7:**
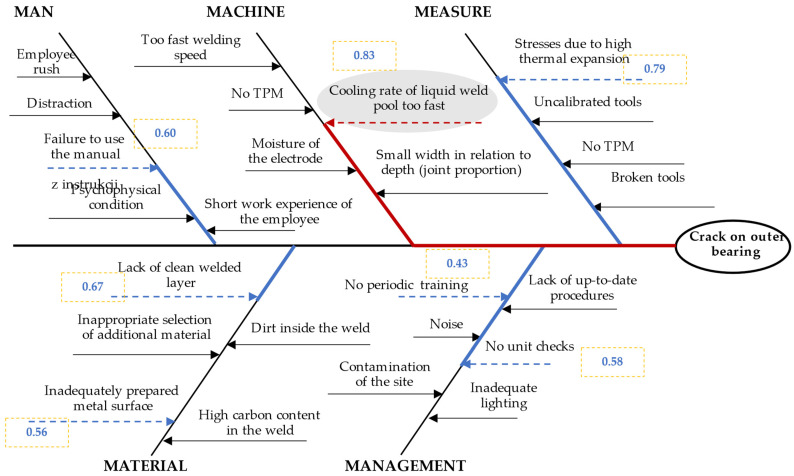
Ishikawa diagram for crack on the outer hull of four-point bearing to choose the main cause.

The main cause of the crack in the outer hull of four-point bearing was the too high cooling rate of the liquid weld pool. This cause had the highest value of the GRA method. This cause was marked in the Ishikawa diagram (Figure 7).

According to the main cause, the ranking of improving actions was created to stabilize the quality of the outer hull of four-point bearing.

**Stage 8.** Creating a ranking of actions as part of stabilizing the quality of the product

At this stage, the second-order causes were sorted. These causes were sorted according to the GRA values. The ranking of second-order causes is presented in Table 5.

As shown in the previous stage, the main cause was the cause with the maximum GRA value, that is, the cooling rate of the liquid weld pool was too high (0.83). According to the proposed concept, improvement actions should be taken for this reason in the first place. According to [42], the post-weld inspection includes the provision of appropriate geometrical dimensions and the development of surface and volume tests. After implementing improvement actions for these causes, one should propose actions to reduce stresses, then clean the surface layer, then the need to follow the instructions and introduce more frequent checks.

## 4. Discussion

Stabilization of the quality of products remains a challenge [6,12,23]. It results from the need to identify the incompatibility of the product, and then its effective verification to determine the main cause of its occurrence [5,7,14,21]. Later, it is possible to identify adequate improvement actions [46,47,48,49]. In addition, for the lack of stable quality of product, the problem consists of a large number of potential causes [50]. On the other hand, determining the most important (main) cause is difficult.

The GRA results were not verified with other MCDM (Multi-Criteria Decision-Making Method) methods. However, we tested other approaches for the possibility of including MCDM in the process of identifying causes of incompatibility [24,25,26,27,28,29]. The test of the combination method MCDM (or FMCDM—Fuzzy Multi-Criteria Decision-Making Method) with the quality management tools was effective in identifying the precise causes of the incompatibilities of product. However, the research so far has focused mainly on analyzing a large number of causes of incompatibility. Therefore, there have been no studies that would combine decision support methods and quality management tools to analyze even a small number of causes of noncompliance. Therefore, this is the main originality of the study, which uses the GRA method. This proposed model has been applied for both a large and small number of causes of incompatibility (even 4 causes). It is important to mention that, applying different MCDM techniques, the ranks are different for the same problem. Therefore, future research will focus on the comparison of existing methods developed [24,25,26,27,28,29] with the new proposed approach.

The problem of simultaneously determining the main cause of the problem was identified in the Polish company. The model was carried out by searching and ranking incompatibilities to identify the incompatibility of cracks on the outer hull of the four-point bearing of alloy 418 (CPW-S 5616). This made it difficult to start effective improvement actions for the incompatibility that generates the largest source of waste. During brainstorming, a team of experts determined the root cause, i.e., state of stress (deformation). Next, the team of experts determined 25 potential causes, which were grouped and visualized in the Ishikawa diagram with the 5M rule. Causes were evaluated on a Likert scale, where the team of experts assessed the impact of causes based on incompatibility. The arithmetic average was calculated from experts’ assessments to determine the weights of potential causes. On the basis of the results, second-order cause was determined. These causes were analyzed using the GRA method. As a result, the ranking of the importance of second-order causes was prepared. The main cause was the cooling rate being too high of the liquid weld pool (0.83). According to the proposed concept, improvement actions should be taken for this reason in the first place. Then, the effectiveness of the actions taken should be verified, and further improvement actions should be taken for the next reason from the ranking.

Therefore, the objective of the study was to develop a new model that supports the stabilization quality of industrial products. After the model, it was shown that it is possible to support the stability quality of industrial products by determining the ranking of causes that have an impact on the incompatibility of the product, where it is realized by determining all potential causes and then by their sequential and constructive reduction to reduce the main causes. The initial test of the model shows that the model can be effective in improving products in the casting industry. On the basis of the proposed model for the problem analyzed, it was possible to show the main cause, which was the high cooling rate of the liquid weld pool. According to the model, for this reason, improvement actions should be taken in the first place. The research conducted so far has not allowed us to indicate this cause as the main cause of the problem. The model allowed us to determine the ranking of other improvement actions. The initial test of the proposed model shows the possibilities of its application to improve the quality of the product in casting processes. It turned out that visualization of incompatibility causes supported by additional decision tools could be the right way to improve the quality of products that occur in special processes. The developed model allows us to show that the ranking of identified causes has essential practical meaning because, according to rule 20/80, reducing the most important causes results in a significant improvement of product quality. This also translates into savings related to, for example, materials. To confirm the results obtained in the preliminary test, it will be necessary to carry out a more extensive investigation for other products and other processes.

The limitation of the proposed model is the need to verify the problem based on the knowledge and experience of the team of experts. This applies to the need for a thoughtful verification of the incompatibility by a properly selected team of experts [9]. Furthermore, the ranking of causes of the problem resulted from individual analysis of problem. Therefore, it may be different in other cases. Additionally, it should be mentioned that the proposed method ignores the statistical dispersion of the quality parameters. The method is complex, although the instruments that can be used in this process support a precise way of right identifying causes of incompatibility. Therefore, it is possible to reduce errors. In future research, they plan to make a computer implementation of this model, and this interface could be relatively simple to use. This study has the objective of showing that it is possible to develop a model to support the process of identifying causes of incompatibility of products.

The verification of the model shows its practical potential for use. In the future, a comparable verification of the model is planned to be performed on various products (including services). Carrying out a large number of verifications (tests) is likely to highlight its advantages and possibly show its limitations. Therefore, future research will focus on developing a computer program for this model. In addition, a dynamic decision-making platform is planned to be developed to make decisions on various types of incompatibilities. As part of future research, it is planned to do more extensive research on the model because the developed model needs tests to show its accuracy in predicting/determining the errors and incompatibilities. However, the results of the model will be influenced by many factors, e.g., the selection of a team of experts, which may make it difficult to compare the results.

## 5. Conclusions

Improving the quality of industrial products requires thoughtful and standardized actions. Therefore, the aim of the article was to develop a new model that supports the stabilization quality of industrial products. It refers to the sequential and coherent way of determining the causes of problems with the quality of products, where the number of verified causes can be equal to even 4. Moreover, it is possible to reduce the causes of the problem from potential causes to the most important causes, which possibly have the most degree of influence on the occurrence of the problem. The model was developed by integrating and used in a sequential way by the selected techniques. Those techniques were quality management tools and decision methods in the fuzzy area, that is, SMART(-ER) method, method of selecting a team of experts, brainstorming (BM), Ishikawa diagram with the 5M rule, technique of importance in the Likert scale, arithmetic average, and Grey Relational Analysis (GRA).

The model was carried out by searching and ranking incompatibilities to often identify incompatibility of cracks on the outer hull of the four-point bearing of alloy 418 alloy (CPW-S 5616). Incompatibility was identified by non-destructive testing (magnetic powder method) in the Polish industry. After testing the model, it was concluded that this model can help support the process of stability, quality of materials, the processes of its formation, and industrial product processes by determining the classification of causes that have an impact on the incompatibility of the product; it is realized by determining all potential causes and then their sequential and constructive reduction to reduce the main causes. It was concluded that the application of the model may help enterprises stabilize the quality of products. Additionally, the model can be used for any type of product, for any incompatibilities, and it can be combined with any quality control. The proposed model can be widely used in many industries. It seems particularly advantageous to use it for special processes. Other areas of application may be industries where a particularly high quality of products is required. It is connected with the necessity of continuous improvement based on identifying and then reducing the causes, and not the effects of noncompliance. Examples of such industries may be the automotive, aviation, etc., industries. The use of the model in relation to various industries requires the execution of appropriate tests.

## Figures and Tables

**Table 1:** Mechanical and physical strengths of 418 alloy. Own study based on [45].

Mechanical and Physical Strengths	Value (21 °C)
Ultimate Tensile Strength (MPa)	965
0.2% yield point (MPa)	760
Elongation (%)	15
Brinell hardness	302–352

**Table 2:** Average weights of potential causes.

Category 5M	No.	Potential Causes	Assessment of Importance	Average Weight
man	1	employee rush	2;3;4;3	3.00
	2	dissociation	2;2;3;1	2.00
	3	not following the manual	5;3;4;5	4.25
	4	psychophysical state	4;2;4;4	3.50
	5	short work experience of the employee	3;2;3;3	2.75
machine	6	too high cutting speed	5;4;5;4	4.50
	7	no TPM	3;3;2;3	2.75
	8	electrode humidity	2;2;3;2	2.25
	9	too high cooling rate of the weld pool liquid	5;5;5;4	4.75
	10	small width in relation to the depth	4;3;4;4	3.75
measure	11	stresses	5;5;2;4	4.00
	12	uncalibrated tool	2;1;2;2	1.75
	13	no TPM	2;3;3;4	3.00
	14	damaged tools	2;1;3;2	2.00
material	15	no cleaned top layer	5;5;3;5	4.50
	16	inappropriate selection of additional material	4;5;2;4	3.75
	17	inadequately prepared metal surface	5;4;4;5	4.50
	18	debris inside the weld	1;2;2;3	2.00
	19	high carbon content in the weld	3;2;2;2	2.25
management	20	no periodic training	5;4;5;4	4.50
	21	noise	2;2;1;2	1.75
	22	environment pollution	2;2;2;2	2.00
	23	lack of up-to-date procedures	2;3;3;2	2.50
	24	no unit checks	5;5;3;5	4.50
	25	inadequate lighting	3;2;3;3	2.75

**Table 3:** Assessment of weights of second-order causes.

Category 5M	No.	Potential Causes	Average Weight
Man	C3	not following the manual	3;5;3;2
Machine	C9	too high cooling rate of the liquid weld pool	5;4;5;4
Measure	C11	stresses	5;4;3;5
Material	C15	no cleaned top layer	4;5;3;3
Material	C17	inadequately prepared metal surface	2;4;4;3
Management	C20	no periodic training	1;3;3;2
Management	C24	no unit checks	2;3;5;2

**Table 4:** Results from GRA to choose the main causes.

5M	No.	Normalization				Grey Relational Coefficient				GRA $\gamma \left(x_{0}^{*},\;x_{i}^{*}\right)$	Ranking	Results
Man	C3	0.50	1.00	0.50	0.25	0.50	1.00	0.50	0.40	0.60	4	
Machine	C9	1.00	0.75	1.00	0.75	1.00	0.67	1.00	0.67	0.83	1	main cause
Measure	C11	1.00	0.75	0.50	1.00	1.00	0.67	0.50	1.00	0.79	2	
Material	C15	0.75	1.00	0.50	0.50	0.67	1.00	0.50	0.50	0.67	3	
Material	C17	0.25	0.75	0.75	0.50	0.40	0.67	0.67	0.50	0.56	6	
Management	C20	0.00	0.50	0.50	0.25	0.33	0.50	0.50	0.40	0.43	7	
Management	C24	0.25	0.50	1.00	0.25	0.40	0.50	1.00	0.40	0.58	5	

where: C3—not following the manual, C9—too high cooling rate of the liquid weld pool, C11—stresses, C15—no cleaned top layer, C17—inadequately prepared metal surface, C20—no periodic training, C24—no unit checks.

**Table 5:** Ranking of activities to stabilize the quality of the bearing housing.

5M	No.	Second-Order Causes	GRA $\gamma \left(x_{0}^{*},\;x_{i}^{*}\right)$	Ranking
Man	C3	not following the manual	0.60	4
Machine	C9	too high cooling rate of the liquid weld pool	0.83	1
Measure	C11	stresses	0.79	2
Material	C15	no cleaned top layer	0.67	3
Material	C17	inadequately prepared metal surface	0.56	6
Management	C20	no periodic training	0.43	7
Management	C24	no unit checks	0.58	5

## Data Availability

Not applicable.
